# No Evidence for the Watching-Eyes Effect on Human Impulsivity

**DOI:** 10.3389/fpsyg.2018.01887

**Published:** 2018-10-08

**Authors:** Asami Shinohara, Shinya Yamamoto

**Affiliations:** ^1^Graduate School of Informatics, Nagoya University, Nagoya, Japan; ^2^Institute for Advanced Study, Kyoto University, Kyoto, Japan; ^3^Graduate School of Intercultural Studies, Kobe University, Kobe, Japan

**Keywords:** watching-eyes effect, impulsivity, time-discounting, reputation seeking, social behavior, altruistic behavior

## Abstract

People often become more altruistic when they think or feel that someone is watching them. Known as the “watching-eyes effect,” this is argued to be caused by the motivation to gain and maintain a positive social reputation as an altruistic individual (the “reputation seeking” mechanism). However, an alternative mechanism underlying the watching-eyes effect could be that people suppress their impulsive tendency to pursue benefit rather than increase their altruism, and this may lead to apparent increases in altruistic tendencies. This “suppressing impulsivity” mechanism is considered intrapersonal rather than socially mediated which is associated with “reputation seeking.” We examined whether the suppressing impulsivity mechanism would be associated with the watching-eyes effect by measuring participants’ impulsivity in the presence of watching-eyes stimuli. In a controlled experiment, we presented life-size pictures of human faces with a direct gaze on a monitor in front of participants taking part in a time-discounting task. Two types of faces, “in-group” (faces of participants’ classmates) and “out-group” (unfamiliar faces) were presented to examine the effect of social attribution. We used a flower picture as a control stimulus. In the time-discounting task, participants chose one of two options: a small amount of money that they could get immediately or a larger amount of money that they could get after a given time interval. The results showed no significant difference in participants’ time-discount rate regardless of the types of stimuli presented during the time-discount task. A post-task questionnaire confirmed that the participants were aware of the presented stimuli and revealed that they paid more attention to the in-group stimuli than to the out-group and flower stimuli, though this difference in attentive states had no effect on their impulsivity during the task. These results suggest that suppressing impulsivity is not a plausible mechanism for the watching-eyes effect. The null effect for the difference between the in-group and out-group stimuli also supports this conclusion. Thus, it is plausible that the watching-eyes effect is caused by the human tendency to boost social reputation and can be mediated by the social relationship with others.

## Introduction

Individuals behave altruistically when they are being watched by a third-party observer. Previous research has shown that people donate more money when they are observed by others than when they are not (Izuma et al., 2010). Interestingly, people become altruistic even when exposed to subtle cues of watching eyes, such as drawings (Haley and Fessler, 2005; Bateson et al., 2006; Francey and Bergmüller, 2012). Bateson et al. (2006) demonstrated that people paid more money into an honesty box used to collect money for drinks at a cafeteria when a cue of being watched (a pictorial stimulus of eyes) was presented compared to a non-social stimulus of flowers. This enhancement of altruistic behavior resulting from perceived observation is called the “watching-eyes effect.”

How does the watching-eyes effect occur? The prevailing explanation is that people behave altruistically in front of watching eyes because of the want to gain or maintain a positive social reputation. Oda et al. (2011) asked participants to complete a dictator game in situations with and without watching-eyes stimuli. After the dictator game, a questionnaire asked what the participants thought about and were concerned about during the game, and how they perceived the experimental situation. The researchers found that it was not fear of punishment from a third party, but concern for the impact on their social reputation from a third party that mediated the increase in the amount of money offered when the participants were exposed to the watching-eyes stimulus (Oda et al., 2011). As the theory of indirect reciprocity (Nowak and Sigmund, 1998, 2005) predicts, when individuals have a positive reputation, the possibility increases that they will be treated favorably by others. Thus, it may be natural that people try to enhance their reputation by displaying altruistic behavior in front of others. Other empirical studies have also supported Oda et al.’s (2011) idea by showing, for example, that people become more cooperative when their identifiable reputations are at stake than when their identities are anonymous (Milinski et al., 2002). These studies suggest that “seeking reputation” might be one plausible mechanism that drives the watching-eyes effect.

At this moment, however, we cannot definitively say that “seeking reputation” is the only mechanism underlying the watching-eyes effect. We propose another possible mechanism, namely, that people might suppress their impulsive tendency to pursue actions for their own benefit when in front of watching eyes, and this may lead to their apparent increase in altruistic behavior. A previous study has shown that individuals’ degree of patience is correlated with their altruistic tendency (Curry et al., 2008). Thus, it is possible that people’s altruistic behavior in front of watching eyes is a consequence of a decrease in selfish impulsivity due to being watched. The seeking reputation hypothesis presumes an increase of altruism, which is more socially mediated. On the other hand, the suppressing impulsivity hypothesis supposes a decrease of selfish impulsivity, which is more intrapersonally mediated. To investigate this possibility, we conducted a time-discounting task that evaluated individuals’ time-discount rate while watching eyes were presented. Time-discount rate refers to the degree to which individuals subjectively discount the value of future rewards as a function of delay in their delivery; this reflects how impulsive individuals are (Curry et al., 2008). We manipulated the social attribution of watching eyes using “in-group” (known people) and “out-group” (strangers) observers. Previous studies have found that people share more money when a recipient is an in-group member than when the recipient is an out-group member (Mifune et al., 2010; Balliet et al., 2014). Based on these studies, it is important to examine what kind of watching eyes stimuli might have an impact on time-discount rates. We assumed that if the watching-eyes effect is mediated socially, the eyes of an in-group member would have a stronger influence on decision making about time-discounting than the eyes of an out-group member, while if the effect was mediated by intrapersonal impulsivity, the in-group and out-group stimuli would not make any difference.

## Materials and Methods

### Participants

The study participants were 43 undergraduate and graduate students (15 male, *M_age_* = 20.6, *SD_age_* = 1.9) from Kobe University in Japan who were recruited from one of four seminars (seminar A, *n* = 12; seminar B, *n* = 6; seminar C, *n* = 8; seminar D, *n* = 17). Participants in the same seminar knew each other and had previous interactions with one another, such as through class discussions. Participants who belonged to different seminars had never met each other. We confirmed this with a post-experiment questionnaire. We defined participants in the same seminar as in-group members, and participants belonging to different seminars as out-group members. Before the experiment, all participants provided written informed consent. The study was approved by Human Ethics Committee of Kobe University, and was conducted in accordance with the 1964 Declaration of Helsinki standards.

### Apparatus and Task

In an experimental room (approximately 3 m × 3 m), we set up two monitors. One monitor (520 mm × 325 mm) showed the picture stimuli; the other monitor (195 mm × 345 mm), presented the time-discounting task via a laptop. We put the stimuli monitor approximately 60 cm in front of each participant. The participants performed a time-discounting task on the laptop using the keyboard. The experimental room was set up for one participant at a time, and there was nothing to suggest that the experimenters were monitoring the participants (e.g., a window or a camera in the room). The participants entered the room alone and were provided headphones to prevent sound interruption during the task.

Following the examples of time-discounting tasks in previous studies (Richards et al., 1997; Shinohara and Yamamoto, 2016), we employed an adjusting-amount procedure. In this time-discounting task, two money amounts appeared on the laptop screen. The amount of money that participants could get after a certain delay (“delayed money”) appeared on the right side of the screen, while the amount of money that participants could get immediately (“immediate money”) appeared on the left side. Participants were told that the monetary amounts were not real rewards but to think of them as though they were, and to choose either delayed money or immediate money by pressing a right or left arrow key on the keyboard. We used the same time-discounting task in a previous study, which found that mirrors have a modest effect on human impulsivity (Shinohara and Yamamoto, 2016), and thus this method has been shown to be valid to measure human impulsivity.

The amount of delayed money was fixed as 10,000 yen or 20,000 yen, while the amount of immediate money paired with the delayed money was systematically adjusted in every trial based on the participants’ previous choices; when a participant chose immediate money (or delayed money), the immediate money amount in the next trial decreased (or increased). This enabled us to find an *indifference point*, which is the subjective value of the delayed money if it were offered immediately (Green et al., 2005; O’Brien et al., 2011). We set three different delay periods for the delayed money; 1 week, 1 month, and 1 year, for a total of six patterns of combinations of delayed money and delay period: “10,000 yen, 1 week,” “10,000 yen, 1 month,” “10,000 yen, 1 year,” “20,000 yen, 1 week,” “20,000 yen, 1 month,” and “20,000 yen, 1 year.” We set the initial amounts of immediate money at the start of the experiment based on results of our preliminary examination so that we could find the indifference points in fewer trials: 8,000 yen as the initial amount of immediate money paired with 1-week delayed 10,000 yen; 7,000 yen against 1-month delayed 10,000 yen; 6,000 yen against 1-year delayed 10,000 yen; 16,000 yen against 1-week delayed 20,000 yen; 14,000 yen against 1-month delayed 20,000 yen; and 12,000 yen against 1-year delayed 20,000 yen. The increase or decrease of the money amount depended on the number of trials: 1,000 yen at 1st–5th trials, 500 yen at 6th–10th trials, 100 yen at 11th–15th trials, and 50 yen at 16th–20th trials. For example, participants might have been given a choice between 10,000 yen given after 1 week and 8,000 yen that they could get immediately. During the 1st–5th trial, if the participant chose the 8,000 yen, on the next trial of “10,000 yen, 1 week” would be 7,000 yen (a decrease of 1,000 yen). On the other hand, if the participant chose 10,000 yen, the amount of immediate money on the next trial would be 9,000 yen (an increase of 1,000 yen). The amount of immediate money was adjusted from trial to trial in this manner until the session finished. We also obtained Reaction Time (RT), which was the duration from the time when the money amount appeared on the screen when the participant pressed either the right or left arrow key. One session included 120 trials; the number of trials for each delayed money and delay period combination was 20 trials. The order of presentation of the six money amounts was random. We used PsychoPy v.1.80.03 to control this task.

On the stimuli monitor, we presented either a picture of a human face or flowers during the task. The human facial pictures were taken as front views with neutral expressions and presented on the monitor in real scale. We prepared two kinds of pictures; one showed a person from the same seminar as the respondent (in-group), and the other showed a person from a different seminar (out-group). We used both male and female faces for in-group and out-group. The order of the conditions was counterbalanced.

After the experiment, a questionnaire was used to ask participants whether they (1) had seen the people in the pictures before; (2) knew the people’s names; (3) attended the same seminar as these people did; and (4) had ever interacted with them. These were “yes” or “no” questions. We asked how much participants cared about the human and flower pictures using a 7-point Likert scale ranging from 1 (*did not care at all*) to 7 (*strongly cared*).

### Procedure

Before the experiment, the participants received instructions and provided consent to participate. We explained that the monetary rewards in the experiment were hypothetical but emphasized that participants should think of them as though they were real money. We also told participants that pictures that would appear on the screen during the task had nothing to do with the task itself. After this explanation, participants entered the experimental laboratory alone and worked on the time-discounting task. After each session, the participants had a short break for 3–5 min outside of the experimental room. After finishing the experimental tasks, the participants completed the questionnaire.

### Data Analysis

Two participants were excluded from the final sample because their average RTs were outliers ( < ± 2 *SD* from the mean), and four participants were excluded because they reported interactions with persons in the out-group. The final sample consisted of 36 participants (23 male). For the data analysis, we calculated the discount rate (*k*) as the index of participants’ impulsivity (O’Brien et al., 2011; Weigard et al., 2014). The standard equation for the calculation of discount rate is *V = A/*(1* + kD*), where *V* is the subjective value of the delayed money (indifference point), *A* is the amount of the delayed money (10,000 yen or 20,000 yen), *D* is the delay period (1 week/ 1 month/ 1 year), and *k* is the discount rate. Higher discount rates were considered to reflect higher impulsivity.

We conducted analyses on the indifference point and the discount rate when the delayed money was 10,000 yen and 20,000 yen separately. We decided to exclude the participants’ data whose indifference point was over 10,000 yen when the delayed money was 10,000 yen, and whose indifference point was over 20,000 yen when the delayed money was 20,000 yen. We did this because it is likely that those participants did not complete the task in a serious manner. The final sample when the delayed money was 10,000 yen consisted of 30 (11 males), and the final sample when the delayed money was 20,000 yen consisted of 27 (12 males). The correlation between the average indifference point and the discount rate was *r* = -0.87, *p* < 0.001 when the delayed money was 10,000 yen, and *r* = -0.92, *p* < 0.001 when the delayed money was 20,000 yen.

## Results

### Preliminary Analysis

We conducted ANOVA on participants’ indifference points when the delayed money was 10,000 yen and 20,000 yen separately and found no significant effect of sex on stimuli (*p*s > 0.27). In further analyses, we regarded the average data of in-group male and in-group female stimuli as an in-group condition data, and the average data of out-group male and out-group female stimuli as an out-group condition data.

### When the Delayed Money Was 10,000 Yen

#### Indifference Point

**FIGURE 1:**
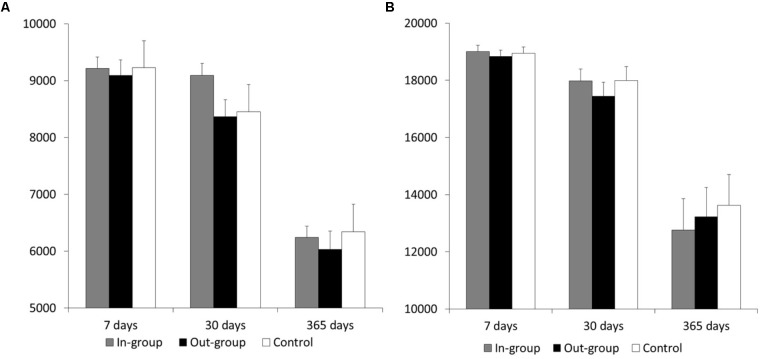
Participants’ indifference points in each condition and each delay period when **(A)** the delayed money was 10,000 yen, and **(B)** the delayed money was 20,000 yen. The error bars indicate *SE*.

The indifference point in each condition is shown in **Figure 1A**. We conducted two-way ANOVA on individuals’ indifference points, using experimental condition (in-group/out-group/flower) and the delay period (1 week/1 month/1 year) as within subject factors. Our aim of this analysis was to find whether there were any differences between the three conditions; that is, to examine whether the main effect of the experimental condition was significant. The interaction between the experimental condition and the delay period was not significant (*F*(4,116) = 0.50, *p* = 0.73, η*^2^* = 0.00). The main effect of the delay period was significant (*F*(2,58) = 42.85, *p* < 0.001, η*^2^* = 0.31). Consistent with previous studies, the indifference points decreased with longer delay periods. However, the analysis found no significant main effect of the experimental condition (*F*(2,58) = 1.79, *p* = 0.18, η*^2^* = 0.00).

#### Discount Rate (k)

**FIGURE 2:**
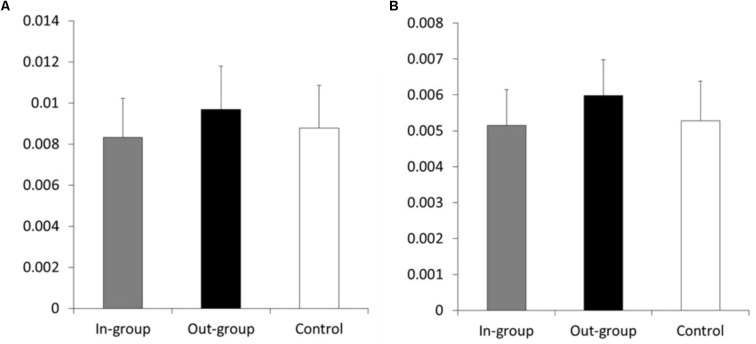
Participants’ discount rates (*k*) in each condition: **(A)** when the delayed money was 10,000 yen, and **(B)** when the delayed money was 20,000 yen. The error bars indicate *SE*.

We conducted one-way ANOVA on the discount rate to test whether the experimental condition influenced participants’ discount rate. There was no significant effect of experimental condition (*F*(2,58) = 1.48, *p* = 0.24, η*^2^* = 0.00) (**Figure 2A**). This is consistent with our prior analysis on the indifference point.

#### Attentiveness to Stimuli

We conducted one-way ANOVA on participants’ attentiveness to the stimuli, that is, how much the participants cared about each specific stimulus (i.e., in-group/out-group/control) as rated on a 7-point Likert scale. The main effect of experimental condition was significant (*F*(2,58) = 8.39, *p* < 0.001, η*^2^* = 0.12). The participants reported that they cared more about the in-group members’ stimuli (*M* = 4.27, *SD* = 1.45) than the out-group stimuli (*M* = 3.07, *SD* = 1.57; *t*(29) = 3.53, *p* < 0.01) and control stimuli (*M* = 3.00, *SD* = 1.76; *t*(29) = 3.30, *p* < 0.01). Participants’ attentiveness to the out-group and the control stimuli were not significantly different (*t*(29) = 0.21, *p* = 0.83).

#### Correlation Between Attentiveness to Stimuli and Discount Rate

To test whether participants’ attentiveness to the stimuli and their discount rate were correlated we conducted a Pearson’s correlation analysis in each condition. There was no significant correlation between two variables in any of conditions (*r* > 0.02, *p* > 0.08), suggesting that participants’ attentiveness was unrelated to their discount rate.

### When the Delayed Money Was 20,000 Yen

Analyses were conducted in the same way as when the delayed money was 10,000 yen.

#### Indifference Point

We conducted two-way ANOVA on participants’ indifference points using experimental condition (in-group/out-group/flower) and delay period (1 week/1 month/1 year) as within subject factors (**Figure 1B**). The main effect of delay period was significant (*F*(2,52) = 32.83, *p* < 0.001, η*^2^* = 0.33). Indifference points decreased as delay periods grew longer. The analysis found that the main effect of experimental condition was not significant (*F*(2,52) = 2.61, *p* = 0.08, η*^2^* = 0.00). The interaction between the experimental condition and the delay period was also significant (*F*(4,104) = 4.38, *p* < 0.01, η*^2^* = 0.00). However, the post-analysis of the simple effects for this interaction did not find any significant differences between the three experimental conditions (*p*s > 0.06).

#### Discount Rate (k)

We conducted one-way ANOVA on the discount rate to test whether experimental condition influenced participants’ discount rate. There was no significant effect of experimental condition (*F*(2,52) = 1.749, *p* = 0.16, η*^2^* = 0.00) (**Figure 2B**). This result was consistent with our prior analysis on the indifference point.

#### Attentiveness to Stimuli

We conducted one-way ANOVA on participants’ attentiveness to the stimuli, which were obtained by 7-point Likert scale, to examine how much the participants cared about a specific stimulus (i.e., in-group/out-group/control). The results were consistent with when the delayed money was 10,000 yen. The main effect of experimental condition was significant (*F*(2,52) = 5.36, *p* < 0.01, η*^2^* = 0.10). The participants reported that they cared more about the in-group members’ stimuli (*M* = 4.17, *SD* = 1.36) than the out-group stimuli (*M* = 3.15, *SD* = 1.61; *t*(26) = 2.75, *p* = 0.03) and the control stimuli (*M* = 3.04, *SD* = 1.76; *t*(26) = 2.65, *p* = 0.03). The participants’ attentiveness to the out-group and control stimuli were not significantly different (*t*(26) = 0.32, *p* = 0.75).

#### Correlation Between Attentiveness to Stimuli and Discount Rate

The analysis of each person’s attentiveness to stimuli and discount rate in each condition found no significant correlation between the two variables in any of the conditions (*r* > 0.06, *p* > 0.53). These results suggest that the participants’ attentiveness was unrelated to their discount rate, and was consistent with when the delayed money was 10,000 yen.

### *Post hoc* Power Analysis

We found no watching-eyes effect on the participants’ indifference point or on the discount rate. In order to eliminate the possibility that our sample size was too small to detect any significant effect of experimental conditions, we conducted *post hoc* power analyses using G^∗^Power 3. The alpha level used for this analysis was *p <* 0.05. Regarding the result of the indifference point, the power exceeded 0.99 in both cases where the delayed money was 10,000 yen and 20,000 yen. In addition, when we conducted the analysis on the result of the discount rate, the power also exceeded 0.99. These findings indicate that our sample size was sufficient to detect any significant effect.

## Discussion

In this study, we investigated whether individuals’ time-discount rate would be influenced by watching eyes to examine the possibility of a suppressing impulsivity mechanism. The results did not support the hypothesis of a suppressing impulsivity mechanism; that is, individuals’ time-discount rate was not affected by watching-eyes stimuli. Participants’ impulsivity did not differ when exposed to in-group members’ eyes and the control picture (flower) or to out-group members’ eyes and the control picture (flower). In addition, we found a null effect for in-group and out-group stimuli, which also did not support a suppressing impulsivity mechanism. Participants’ time-discount rates were not significantly different between being shown pictures of in-group or out-group members. In contrast, we found that individuals cared more about the watching-eyes stimuli of in-group members than the stimuli of out-group members or the control stimulus. However, this higher attentiveness to the in-group members’ watching eyes was not related to their time-discount rate.

We found no effect of watching eyes on participants’ time-discount rates. A previous study suggested that the effect of watching eyes can be found only when people engage in social interaction tasks, such as the dictator game, but not in personal decision-making tasks (Baillon et al., 2013). Our result is consistent with this study; people’s time-discount rates, which were measured by the personal decision-making task, were not influenced by watching-eyes stimuli. Given this, not an intra-personal mechanism but an interpersonal mechanism may plausibly explain the watching-eyes effect. Previous studies found that people care about their reputation in the eyes of a third party (and do not suppress their impulsive temperament to pursue behaviors to their own benefit) when they are being watched, and therefore display altruistic behavior (Oda et al., 2011). Some studies have suggested that decision making regarding altruistic behavior (e.g., donating) when individuals are being watched involves their mentalizing system (Amodio and Frith, 2006; Izuma, 2012). These findings support other studies that suggest that when individuals are being watched by someone, they care how these people think about their behavior, and consider whether their behavior would enhance or diminish their reputation. Thus, it is plausible that the watching-eyes effect is caused by a human tendency to value social reputation. This can be mediated by social relationships with others in terms of the watching-eyes stimuli; exploring this effect further should be a target for future research. At this moment, the “reputation seeking mechanism” seems to be a plausible mechanism, but it is necessary to reveal whether there is another mechanism of the watching-eyes effect. For example, being watched may arouse individuals’ self-awareness, resulting in displaying more prosocial behavior (Abbate et al., 2006; Abbate and Ruggieri, 2008).

There may be other reasons why we found no effect of watching eyes on human impulsivity. Firstly, the participants were exposed to the watching eyes for a relatively long time (*M* = 7.17 min, *SD* = 2.09). Sparks and Barclay’s (2013) study suggested that a short, rather than long, exposure to eyes stimuli induces the watching-eyes effect. They conducted an experiment and meta-analysis of experimental data that utilized the dictator game during watching-eyes exposure. They found the watching-eyes effect in participants’ altruistic tendency when watching-eyes stimuli were shown “shortly” (i.e., eye stimuli are suddenly visible or attention is drawn to them shortly; Sparks and Barclay, 2013); however, such effect was not confirmed in “prolonged exposure” conditions (i.e., eye stimuli are visible and in the participant’s line of vision for several minutes; Sparks and Barclay, 2013; e.g., *M* = 3.46 min in their experiment). These findings suggest that the watching-eyes effect is an unconscious response to false social cues (Sparks and Barclay, 2013). Our long exposure might have altered participants’ awareness of, and reactions to, being watched, which might have led to the null effect of watching-eyes on impulsivity. However, there have also been several studies which found the watching-eyes effect even in long stimulus-exposure conditions (e.g., Bateson et al., 2006), and thus we do not think that this is the only critical point. The effect of manipulating the exposure time of watching-eyes on impulsivity is worthy of future study. Second, our watching eyes stimuli might not be strong enough to change people’s decision, given that the watching-eyes effect on human generosity is relatively weak (Northover et al., 2017). Though we could not fully eliminate this possibility, we think that our stimuli were stronger than some of the other stimuli, such as schematic eye drawings (e.g., Haley and Fessler, 2005) that were effective in inducing the watching-eyes effects in previous studies. The stimuli we used in this study contained social information, such as social group, and thus could plausibly simulate more realistic situations compared to the stimuli used in previous studies. Neverthless, further research is required to examine whether the conventional watching-eyes stimuli used in previous studies could influence our participants’ time-discounting in order to make a direct comparison.

## Conclusion

In conclusion, our study shows that watching-eyes do not influence human impulsivity, even though we manipulated the social attribution of watching-eyes, which may support the notion that people behave altruistically when they are being watched because they are concerned about their social reputation. This study is the first to examine the underlying mechanism of the watching-eyes effect, and shows that the reputation-seeking mechanism remains a plausible explanation for this effect. This study also provides some ideas for future research on the mechanisms of the watching-eyes effect, which is probably a mental process that is unique to humans.

## Author Contributions

AS and SY contributed to the design and implementation of the research and wrote the manuscript. AS conducted the experiment.

## Conflict of Interest Statement

The authors declare that the research was conducted in the absence of any commercial or financial relationships that could be construed as a potential conflict of interest.

## References

[B1] Abbate C. S. Isgrò A. Wicklund R. A. Boca S. (2006). A field experiment on perspective-taking, helping, and self-awareness. *Basic Appl. Soc. Psychol.* 28 283–287. 10.1207/s15324834basp2803-7

[B2] Abbate C. S. Ruggieri S. (2008). A beggar, self-awareness and willingness to help. *Curr. Psychol. Lett.* 24 98–107.

[B3] Amodio D. M. Frith C. D. (2006). Meeting of minds: the medial frontal cortex and social cognition. *Nature Rev. Neurosci.* 7 268–277. 16552413 10.1038/nrn1884

[B4] Baillon A. Selim A. van Dolder D. (2013). On the social nature of eyes: the effect of social cues in interaction and individual choice tasks. *Evol. Hum. Behav.* 34 146–154. 10.1016/j.evolhumbehav.2012.12.001

[B5] Balliet D. Wu J. De Dreu C. K. W. (2014). Ingroup favoritism in cooperation: a meta-analysis. *Psychol. Bull.* 140 1556–1581. 10.1037/a0037737 25222635

[B6] Bateson M. Nettle D. Roberts G. (2006). Cues of being watched enhance cooperation in a real-world setting. *Biol. Lett.* 2 412–414. 10.1098/rsbl.2006.0509 17148417 PMC1686213

[B7] Curry O. S. Price M. E. Price J. G. (2008). Patience is a virtue: cooperative people have lower discount rates. *Pers. Indiv. Diff.* 44 780–785. 10.1016/j.paid.2007.09.023

[B8] Francey D. Bergmüller R. (2012). Images of eyes enhance investments in a real-life public good. *PLoS One* 7:e37397. 10.1371/journal.pone.0037397 22624026 PMC3356250

[B9] Green L. Myerson J. Macaux E. W. (2005). Temporal discounting when the choice is between two delayed rewards. *J. Exp. Psychol.* 31:1121. 10.1037/0278-7393.31.5.1121 16248754

[B10] Haley K. J. Fessler D. M. T. (2005). Nobody’s watching? Subtle cues affect generosity in an anonymous economic game. *Evol. Hum. Behav.* 26 245–256. 10.1016/j.evolhumbehav.2005.01.002

[B11] Izuma K. (2012). The social neuroscience of reputation. *Neurosci. Res.* 72 283–288. 10.1016/j.neures.2012.01.003 22285602

[B12] Izuma K. Saito D. N. Sadato N. (2010). Processing the incentive for social approval in the ventral striatum during charitable donation. *J. Cogn. Neurosci.* 22 621–631. 10.1162/jocn.2009.21228 19320552

[B13] Mifune N. Hashimoto H. Yamagishi T. (2010). Altruism toward in-group members as a reputation mechanism. *Evol. Hum. Behav.* 31 109–117. 10.1016/j.evolhumbehav.2009.09.004

[B14] Milinski M. Semmann D. Krembeck H. J. (2002). Reputation helps solve the “tragedy of the commons.” *Nature* 415 424–426. 10.1038/415424a 11807552

[B15] Northover S. B. Pedersen W. C. Cohen A. B. Andrews P. W. (2017). Artificial surveillance cues do not increase generosity: two-meta-analyses. *Evol. Hum. Behav.* 38 144–153. 10.1016/j.evolhumbehav.2016.07.001

[B16] Nowak M. A. Sigmund K. (1998). Evolution of indirect reciprocity by image scoring. *Nature* 393 573–577. 10.1038/31225 9634232

[B17] Nowak M. A. Sigmund K. (2005). Evolution of indirect reciprocity. *Nature* 437 1291–1298. 10.1038/nature04131 16251955

[B18] O’Brien L. Albert D. Chein J. Steinberg L. (2011). Adolescents prefer more immediate rewards when in the presence of their peers. *J. Res. Adolesc.* 21 747–753. 10.1111/j.1532-7795.2011.00738.x

[B19] Oda R. Niwa Y. Honma A. Hiraishi K. (2011). An eye-like painting enhances the expectation of a good reputation. *Evol. Hum. Behav.* 32 166–171. 10.1016/j.evolhumbehav.2010.11.002

[B20] Richards J. B. Mitchell S. H. de Wit H. Seiden L. S. (1997). Determination of discount functions in rats with an adjusting-amount procedure. *J. Exp. Anal. Behav.* 67 353–366. 10.1901/jeab.1997.67-353 9163939 PMC1284611

[B21] Shinohara A. Yamamoto S. (2016). Mirrors have a modest effect on human impulsivity. *Lett. Evol. Behav. Sci.* 7 25–28.

[B22] Sparks A. Barclay P. (2013). Eyes images increase generosity, but not for long: the limited effect of a false cue. *Evol. Hum. Behav.* 34 317–322. 10.1016/j.evolhumbehav.2013.05.001

[B23] Weigard A. Chein J. Albert D. Smith A. Steinberg L. (2014). Effects of anonymous peer observation on adolescents’ preference for immediate rewards. *Dev. Sci.* 17 71–78. 10.1111/desc.12099 24341973 PMC3869036

